# Maternal voice reduces procedural pain in neonates: A meta-analysis of randomized controlled trials

**DOI:** 10.1097/MD.0000000000033060

**Published:** 2023-03-24

**Authors:** Lingwen Jin, Jing Zhang, Xin Yang, Hui Rong

**Affiliations:** a NICU, Children’s Hospital of Nanjing Medical University, Nanjing, China.

**Keywords:** care, maternal voice, neonates, nursing, procedural pain

## Abstract

Procedural pain management is a very important part in the clinical care of neonates. We aimed to conduct a meta-analysis to evaluate the effects of maternal voice on neonatal procedural pain, to provide insights to the clinical pain care of neonates.

**Methods:**

Two researchers independently searched PubMed, EMBASE, The Cochrane Library, CINAHL, Web of Science, China Biomedical Literature Database, China National Knowledge Infrastructure, Wanfang and Weipu Database for randomized controlled trials (RCTs) involving the effects of maternal voice on the procedural pain of neonates up to October 25, 2022. Two investigators screened the literature based on the inclusion and exclusion criteria and evaluated the methodological quality of the inclusion study. RevMan 5.3 software was used for the meta-analysis.

**Results:**

A total of 8 RCTs with a total of 584 neonates were included. Our meta-analysis indicated that maternal voice reduces the pain score (SMD = −0.60, 95% CI: −0.81 to −0.39) and heart rate (SMD = −0.81, 95% CI: −1.44 to −0.18) and increases the comfort level (SMD = −0.47, 95% CI: −0.83 to −0.11) and blood oxygen saturation (SMD = 0.70, 95% CI: 0.03–1.38) during procedure (all *P* < .05). Moreover, maternal voice reduces the pain score (SMD = −0.58, 95% CI: −0.88 to −0.28) and heart rate (SMD = −0.44, 95% CI: −0.75 to −0.12) and increases the blood oxygen saturation (SMD = 0.41, 95% CI: 0.00 to −0.82) after procedure (all *P* < .05). No publication biases were detected by the funnel plots and Egger tests (all *P* > .05).

**Conclusion:**

Maternal voice is beneficial to reduce procedural pain and improve the physiological indicators in neonates. It is still necessary to conduct high-quality, large sample studies in the future to further elucidate the effect of maternal voice on neonatal pain care.

## 1. Introduction

Pain is a common yet serious adverse stimulus faced by neonates in the neonatal intensive care unit (NICU).^[1]^ A previous study^[2]^ has shown that NICU neonates have experienced an average of 7.5 to 17.3 times of operational pain every day in the first 2 weeks after hospitalization. A Chinese survey^[3]^ has shown that the median number of pain causing operations received by children every day was 20. It has been reported that neonates have anatomical, neurochemical, and functional bases for pain perception, and neonates have the ability to respond to pain.^[4]^ Studies^[5]^ have shown that premature infants need to stay in the NICU for monitoring and treatment after birth, and receive various painful operations such as arteriovenous puncture, foot blood collection, and tracheal intubation. Many repeated pain stimuli will cause stress reactions to the body, leading to a series of short-term and long-term adverse effects on neonates.^[6–8]^

Non-drug interventions play an important role in neonatal pain management due to their simplicity and less adverse effects. In the sound environment of the fetus, the mother’s voice plays a leading role.^[9]^ The mother’s voice is crucial to the development of neural and cognitive behavior of the late fetus, and can enable premature infants to show a relaxed and focused behavioral state.^[10]^ In the exploration of relieving the procedural pain of infants, the maternal voice, as a new intervention method, has received the attention of scholars at home and abroad in recent years. The mother’s voice is a non-drug intervention measure, which refers to playing the recorded mother’s voice during the painful operation of children. Newborn babies with gestational age of more than 32 weeks can recognize, perceive and respond to the mother’s voice.^[11]^ Previous studies^[12,13]^ have shown that the mother’s voice is a benign stimulus, which can stabilize the physiological state of the newborn, accelerate its feeding process, and promote its growth and development. In recent years, there have been studies focusing on the effect of mother’s voice on relieving neonatal operational pain, but most of them are at the stage of clinical exploration of small samples, and the research results are still controversial. Therefore, this study aimed to evaluate the effect of maternal voice on neonatal procedural pain, to provide scientific evidence for clinical nursing care of neonates.

## 2. Methods

This systematic review was performed and reported in compliance with the preferred reporting items for systematic reviews and meta-analyses statement.^[14]^ Ethical approval and consent to participate was not necessary.

### 2.1. Literature retrieval strategy

The two researchers independently searched PubMed, EMBASE, The Cochrane Library, CINAHL, Web of Science, China Biomedical Literature Database, China National Knowledge Infrastructure, Wanfang Database and Weipu Database for randomized controlled trials (RCTs) on the effects of maternal voice on the procedural pain of neonates. The retrieval time limit was from the establishment of the database to October 25, 2022. This meta-analysis used a combination of subject words and free words for literature retrieval. The retrieval strategies of this meta-analysis were (“newborn” OR “neonate” OR “premature infant” OR “very low birth weight infant” OR “ultra-low birth weight infant” OR “infant”) and (“voice” OR “mother voice” OR “maternal voice”) and (“pain”). At the same time, we traced the references of included RCTs and related reviews to cover more relevant articles.

### 2.2. Inclusion and exclusion criteria

The inclusion criteria of RCTs in this meta-analysis were as follows: RCT study design; The study population were neonates who did not receive sedative or analgesic drugs before procedure, including premature infants and term neonates; The intervention measure was to give the mother’s voice during the operation of neonates in the experimental group. The mother’s voice can be either a live voice or a recorded voice including the mother’s heartbeat, story-telling, songs singing and speaking. The control group only accepted routine clinical procedures during this process; and The RCT reported the corresponding results data, including pain levels in newborns rated by neonatal infant acute pain assessment scale, premature infant pain profile, neonatal infant pain scale, premature infant comfort scale et al And the physiological indicators such as heart rate or blood oxygen saturation were reported.

The literature exclusion criteria for this meta-analysis included: animal experiments, cases, reviews, and expert opinions; repeatedly published literature; incomplete data or full text literature that could not be obtained.

### 2.3. Literature screening and data extraction

According to the inclusion and exclusion criteria of the literature, two researchers independently completed the preliminary and further screening of the literature by reading the title, abstract and full text. The first two researchers read the two articles to be included in the literature together, determined that the data could be extracted independently, and then cross checked the data after completion. In case of differences, the two researchers negotiated, or the third researcher arbitrated. The data extracted from this meta-analysis included: first author, published years, study setting, sample size, procedure details, intervention measures, control measures and outcome indicators.

### 2.4. Quality evaluation of included RCTs

The Cochrane Collaboration’s risk of bias tool^[15]^ was adopted to evaluate the methodological quality and risk of bias of included studies. The evaluation contents of this tool include random sequence generation, allocation hiding, blind method, integrity of result data, evaluation method of outcome indicators, data analysis method, and other risk sources. The evaluation process was conducted independently by two researchers. In case of disagreement, both parties discussed, or the third researcher arbitrated.

### 2.5. Statistical methods

This meta-analysis used RevMan 5.3 software for data analysis. Chi square test was used for heterogeneity test. If *P* > .1, *I*^2^ ≤ 50%, indicating that there was no significant statistical heterogeneity, and fixed effect model was used for analysis; If *P* ≤ .1 and *I*^2^ > 50%, indicating that there was statistical heterogeneity, then a random effect model was used. The continuous measurement data were calculated by the standardized mean difference (SMD), and the 95% confidence interval (CI) was used to represent the size of the combined effect of meta-analysis. Publication bias was assessed by funnel plots, and the asymmetry was rated by Egger regression test. Besides, we conducted sensitivity analyses to evaluate the influence of single one study on the synthesized results. *P* < .05 indicated that the difference was statistically significant between two groups.

## 3. Results

### 3.1. RCTs inclusion

**Figure 1.**
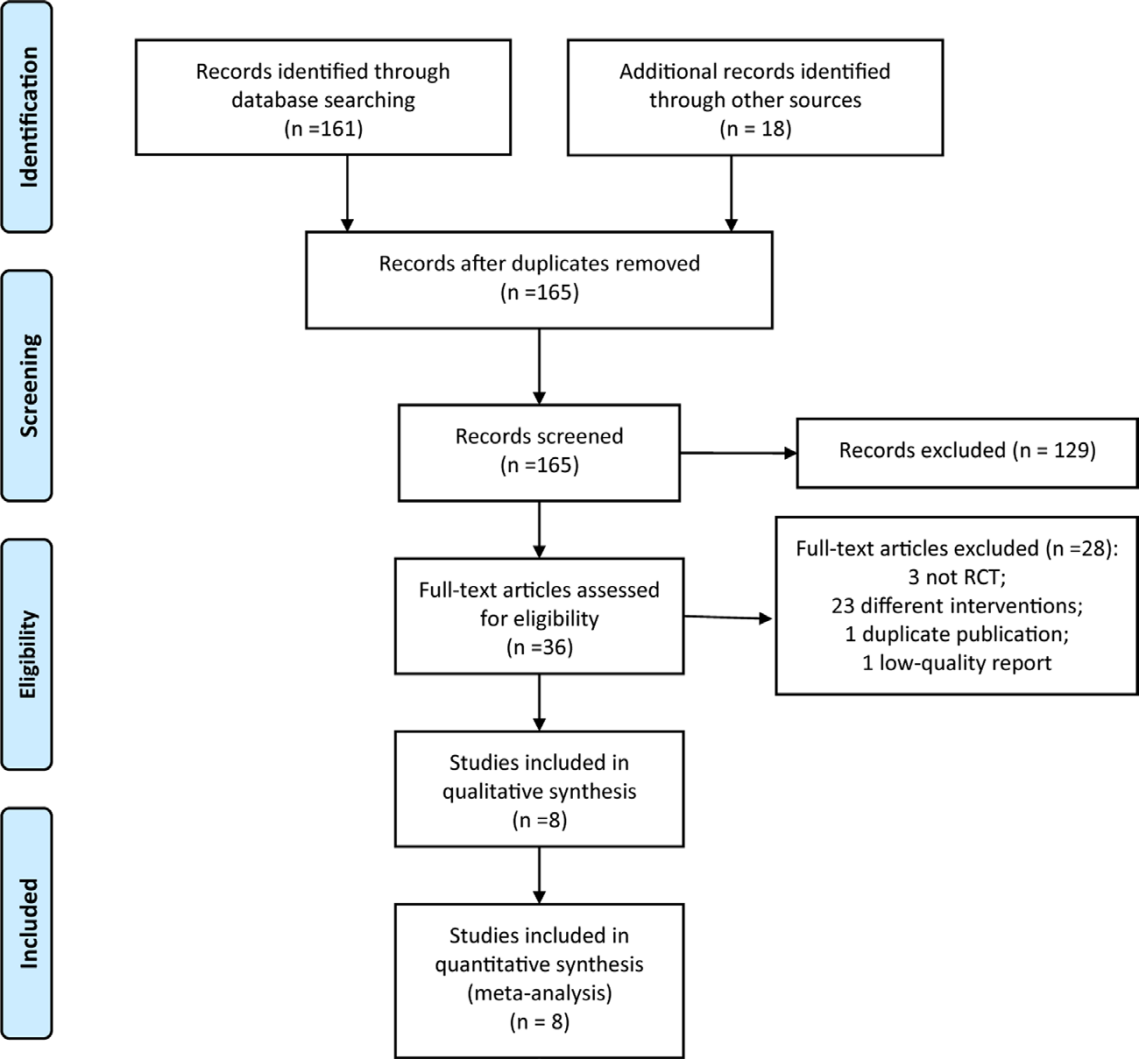
PRISMA flow diagram of study selection. PRISMA = preferred reporting items for systematic reviews and meta-analyses, RCTs = randomized controlled trials.

A total of 161 articles were obtained through preliminary searches of the databases, and 18 articles were obtained through other ways. After layer-by-layer screening, 8 RCTs^[16–23]^ were finally included. The flow chart of literature retrieval and screening is shown in Figure 1.

### 3.2. The characteristics and quality of included RCTs

Of the included 8 RCTs, a total of 584 neonates were included, 292 neonates underwent maternal voice intervention and routine care, respectively. There were no statistically significant differences between the experimental group and the control group in terms of baseline data and pain scores before operation, which was comparable. The basic information of RCTs included are presented in Table 1.

**Table 1:** The characteristics of included studies.

Study ID	Country	Sample size		Procedure	Interventions		Outcomes
		Maternal voice group	Control group		Maternal voice group	Control group	
Alemdar 2018a	Turkey	32	30	Endotracheal suctioning	Mother’s heartbeat; The intervention time ranged from 1 min before operation to 15 min after operation or the recording ended; The volume was 45 dB	Routine care	Degree of pain (PIPP score) and comfort (PICS score)
Alemdar 2018b	Turkey	30	32	Peripheral vein puncture	The mother’s voice, the intervention time from 15 min before puncture to 15 min after puncture, the volume was 45 dB	Routine care	Degree of pain (PIPP score) and comfort (PICS score)
Azarmnejad 2015	Iran	30	30	Arterial blood collection	Mother’s voice, intervention time from 10 min before puncture to 10 min after puncture or the end of recording; The volume was 50–60 dB	Routine care	Degree of pain (NIPS score)
Azarmnejad 2017	Iran	30	30	Peripheral vein puncture	Mother’s voice, intervention time from 10 min before puncture to 10 min after puncture or the end of recording; The volume is 50–60 dB	Routine care	Physiological indicators: including heart rate, blood oxygen saturation, respiration, systolic blood pressure and diastolic blood pressure.
Chen 2018	China	58	58	Peripheral vein puncture	Playing the recorded mother’s voice, which could be mother talking, reading stories, singing children’s songs; The intervention time was from 2 min before venipuncture to the end of the operation or the neonate returned to calm state; The volume was 50–60 dB	Swaddled package	Pain degree (NIA-PAS score), physiological indicators including heart rate, blood oxygen saturation, the beginning and duration of the first painful face
Chen 2019	China	35	37	Heel blood collection	Playing the recorded mother’s voice, which could be mother’s speech, storytelling and singing; The intervention time was from 1 min before blood collection to the end of operation; The volume is 50 dB	Routine care	Pain degree (PIPP-R score). Physiological indexes including heart rate, blood oxygen saturation, and crying of newborn
Erdoğan 2020	Turkey	30	30	Vascular access, blood collection or aspiration	Playing the recorded mother’s voice, which could be mother’s speech, storytelling and singing	Routine care	Pain level, heart rate and oxygen saturation
Sun 2022	China	47	45	Radial artery puncture	Play the prerecorded mother’s voice from 1 min before blood collection to the end of operation; The volume was 40–50 dB	Routine care	Degree of pain (NIPS score), physiological indexes including heart rate, blood oxygen saturation, and crying of newborn

NIAPAS = neonatal infant acute pain assessment scale, NIPS = neonatal infant pain scale, PIPP = premature infant pain profile, PICS = premature infant comfort scale.

**Figure 2.**
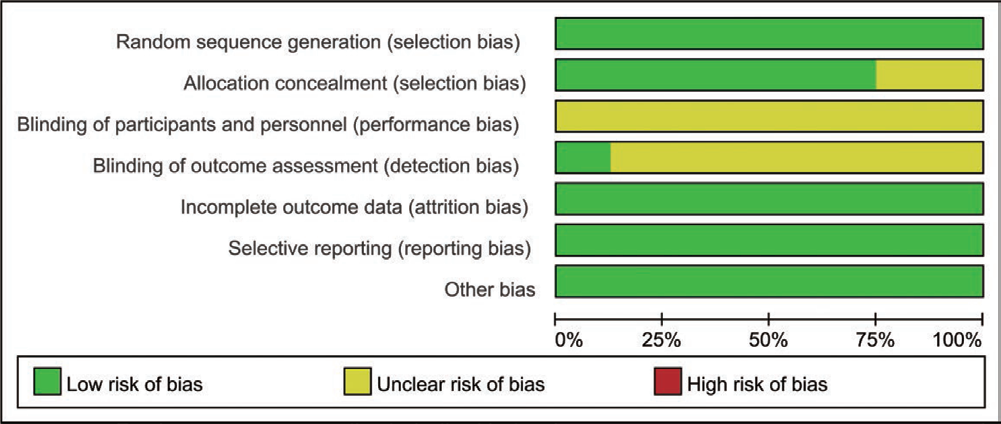
Risk of bias graph of included RCTs. RCTs = randomized controlled trials.

**Figure 3.**
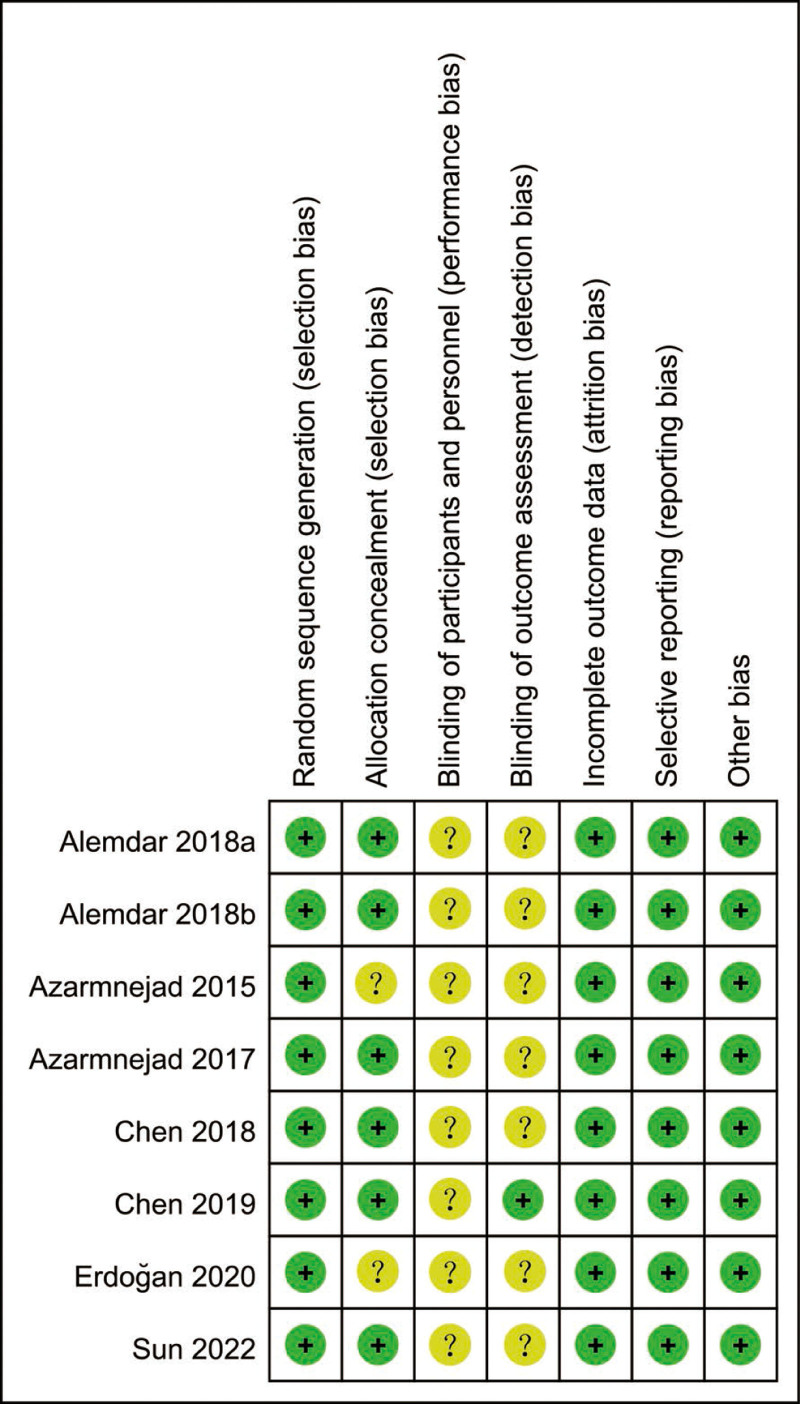
Risk of bias summary of included RCTs. RCTs = randomized controlled trials.

The risk of bias for included RCTs were presented in Figures 2 and 3. All included 8 RCTs mentioned the methods to generate randomization sequence. 2 RCTs did not report the methods of allocation concealment. The blind design of participants, intervention and the outcome assessment remained unclear. No other biases were found amongst the included RCTs.

### 3.3. Meta-analysis

#### 1.3.3. Pain score during procedure.

**Figure 4.**
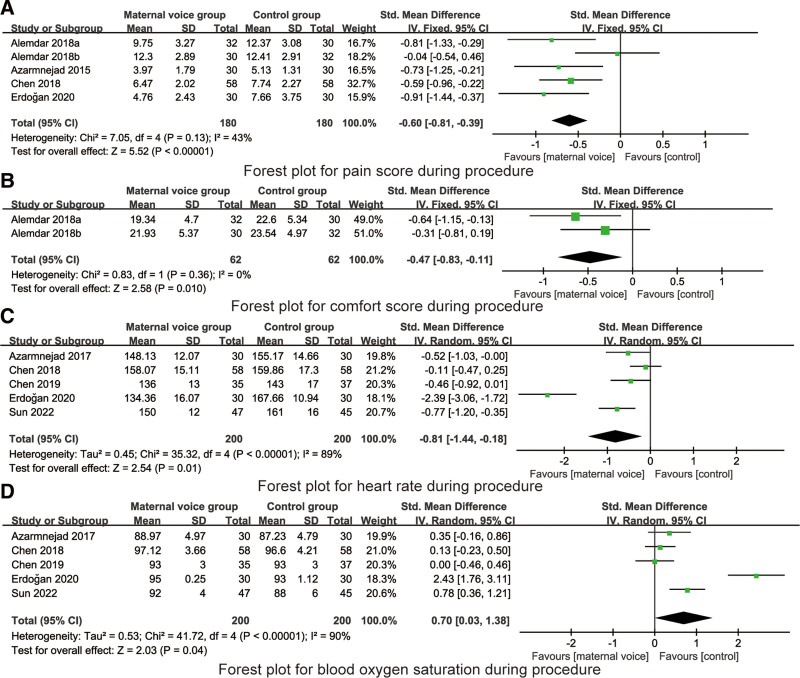
Forest plots for the pain score, comfort score, heart rate and blood oxygen saturation during procedure. CI = confidence interval.

5 RCTs reported the pain score during procedure. There was no heterogeneity on the pain score during procedure (*I*^2^ = 43%, *P* = .13), so the fixed model was used for data analysis. Meta-analysis indicated that maternal voice reduced the pain score during procedure (SMD = −0.60, 95% CI: −0.81 to −0.39, *P* < .001, Fig. 4A).

#### 2.3.3. Comfort score during procedure.

2 RCTs reported the comfort score during procedure. There was no heterogeneity on the pain score during procedure (*I*^2^ = 0%, *P* = .36), so the fixed model was used for data analysis. Meta-analysis indicated that maternal voice increased the comfort level during procedure (SMD = −0.47, 95% CI: −0.83 to −0.11, *P* = .01, Fig. 4B).

#### 3.3.3. heart rate during procedure.

5 RCTs reported the heart rate during procedure. There was heterogeneity on the heart rate during procedure (*I*^2^ = 89%, *P* < .001), so the random model was used for data analysis. Meta-analysis indicated that maternal voice reduced the heart rate during procedure (SMD = −0.81, 95% CI: −1.44 to −0.18, *P* < .001, Fig. 4C).

#### 4.3.3. Blood oxygen saturation during procedure.

5 RCTs reported the blood oxygen saturation during procedure. There was heterogeneity on the blood oxygen saturation during procedure (*I*^2^ = 90%, *P* < .001), so the random model was used for data analysis. Meta-analysis indicated that maternal voice increased the blood oxygen saturation during procedure (SMD = 0.70, 95% CI: 0.03–1.38, *P* = .04, Fig. 4D).

#### 5.3.3. Pain score after procedure.

**Figure 5.**
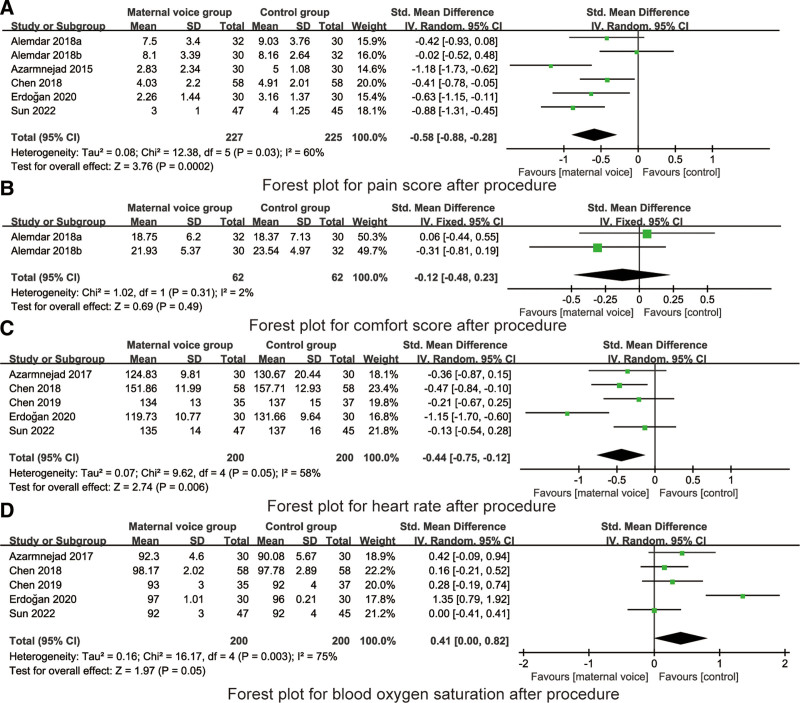
Forest plots for the pain score, comfort score, heart rate and blood oxygen saturation after procedure. CI = confidence interval.

6 RCTs reported the pain score after procedure. There was no heterogeneity on the pain score after procedure (*I*^2^ = 60%, *P* = .03), so the random model was used for data analysis. Meta-analysis indicated that maternal voice reduced the pain score after procedure (SMD = −0.58, 95% CI: −0.88 to −0.28, *P* = .03, Fig. 5A).

#### 6.3.3. Comfort score after procedure.

2 RCTs reported the comfort score after procedure. There was no heterogeneity on the pain score after procedure (*I*^2^ = 2%, *P* = .31), so the fixed model was used for data analysis. Meta-analysis indicated that there was no significant difference in the comfort score after procedure between maternal voice and control group (SMD = −0.12, 95% CI: −0.48 to 0.23, *P* = .49, Fig. 5B).

#### 7.3.3. Heart rate after procedure.

5 RCTs reported the heart rate after procedure. There was heterogeneity on the heart rate after procedure (*I*^2^ = 58%, *P* = .05), so the random model was used for data analysis. Meta-analysis indicated that maternal voice reduced the heart rate after procedure (SMD = −0.44, 95% CI: −0.75 to −0.12, *P* = .006, Fig. 5C).

#### 8.3.3. Blood oxygen saturation after procedure.

5 RCTs reported the heart rate after procedure. There was heterogeneity on the heart rate during procedure (*I*^2^ = 75%, *P* = .003), so the random model was used for data analysis. Meta-analysis indicated that maternal voice reduced the heart rate after procedure (SMD = 0.41, 95% CI: 0.00 to −0.82, *P* = .05, Fig. 5D).

### 3.4. Publication bias and sensitivity analysis

**Figure 6.**
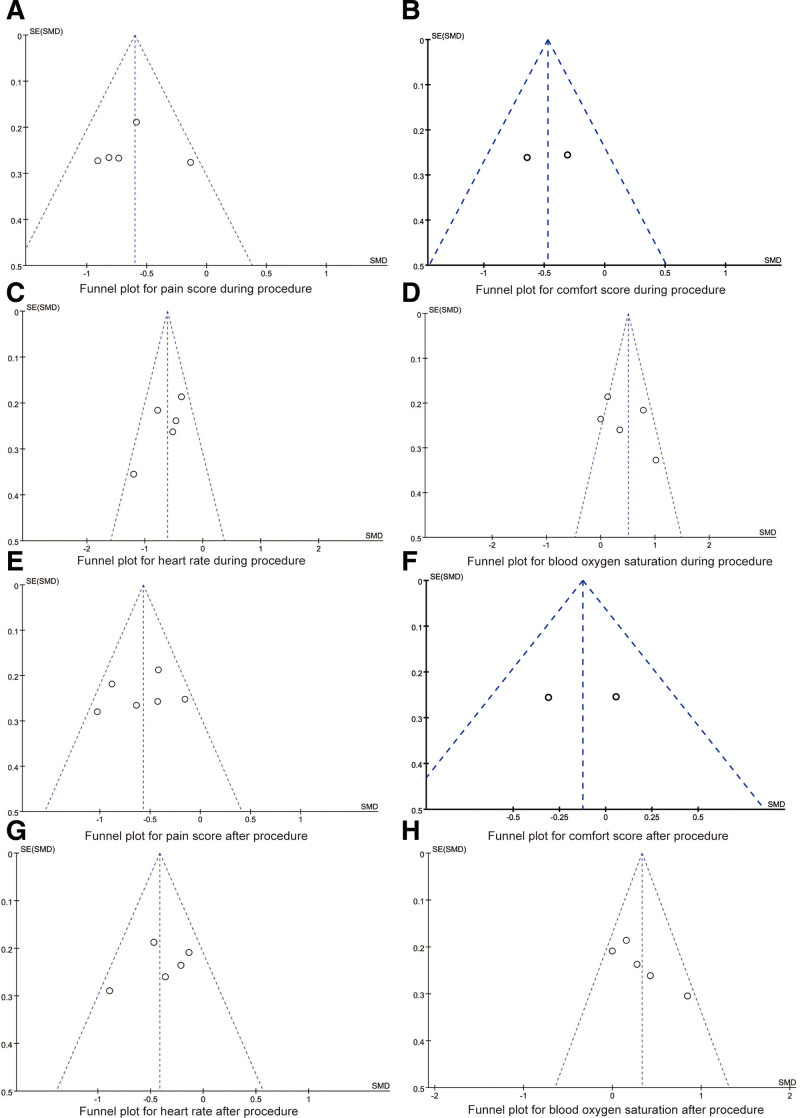
Funnel plots for synthesized outcomes. SMD = standardized mean difference.

Funnel plots for synthesized outcomes are showed in Figure 6. The dots in the funnel plots were evenly distributed, and no publication bias was detected by Egger tests (all *P* > .05).

We excluded the RCT on every result one by one to check whether the results changed, and the overall results did not change by excluding any included RCTs.

## 4. Discussion

Pain is a subjective feeling of discomfort and a comprehensive reaction process of feeling, emotion, cognition and behavior. Epidemiological investigation^[24]^ shows that NICU newborns have to undergo a lot of painful operations during hospitalization. For a long time, due to the lack of language expression ability and cognitive limitations of the newborn, neonatal pain is often ignored or underestimated, and has not been properly treated in a timely manner.^[25]^ Studies^[26,27]^ have shown that the fetus has formed rich sensory nerves in the second trimester of pregnancy. The newborn’s perception of pain is even more intense and lasting than that of adults.^[28]^ Repeated pain stimulation will change the endocrine system of premature infants in the short term, cause growth retardation and early neurodysplasia, and affect pain perception, cognition and movement in the long term.^[29,30]^ The application of drugs to intervene the pain of premature infants lacks accurate research on the safety and long-term effects. Therefore, it is particularly important to explore safe, convenient and effective non-drug interventions to reduce neonatal pain level. The results of this meta-analysis show that the maternal voice can help to reduce the pain degree during the painful operation, reduce the rising level of heart rate, and improve comfort and blood oxygen saturation. At the same time, it is also helpful to reduce the pain score and the heart rate level after the painful operation.

Studies^[31,32]^ have shown that at 24 weeks of gestation, the fetal cochlea and peripheral sensory organs have been developed and can receive sound information. The earliest and most vocal stimuli exposed in the fetal period are the mother’s voice and heartbeat. The pregnant woman’s daily language and her own body voice are enough to stimulate the newborn’s hearing development.^[33]^ It’s been reported that newborns can recognize their mothers’ voices.^[34]^ The brain of the newborn produces different blood flow changes under the stimulation of the mother’s voice and the female nurse’s voice, both of which can cause the blood flow in the left frontal lobe to increase, but the nurse’s voice can cause the blood flow in the right frontal lobe to increase, while the mother’s voice has no such effect.^[35,36]^ The nurse’s voice stimulation may be supplemented after the results and reasons, which needs further investigations in future studies. As a kind of auditory stimulus that the fetus is already familiar with in the womb, compared with the unfamiliar noise in NICU, the mother’s voice can provide a comfortable and relaxing environment for the neonates.^[37,38]^ Some studies^[39,40]^ have pointed out that NICU children should be able to contact their parents’ voices at the bedside to increase parent-child interaction.

Neonatal operational pain is very common, and non-drug intervention is an important measure to alleviate neonatal pain. Common interventions include kangaroo care, NNS, breastfeeding and sensory stimulation. Sensory stimulus includes tactile stimulus, auditory stimulus, language stimulus, visual stimulus and taste stimulus, which can be used together. It is recommended to be applied to all newborns receiving invasive operation. As a means of sensory stimulation, the mother’s voice can not only relieve some slight operational pain, but also provide a link between mother and neonates compared with other sound stimulation means such as brainwave music or white noise.^[41,42]^ Beauchemin et al^[39]^ analyzed the activity of the cerebral cortex of the neonates when exposed to the voice of the mother and strangers, and the results have showed that the newborn is more active in processing the mother’s voice, and the mother’s voice stimulation will trigger the activity of the early language related processing brain regions. A study^[43]^ on 20 premature infants with cumulative observation time of 13,680 minutes has shown that the heart rate of premature infants in the nursing period exposed to the mother’s voice stimulation is significantly lower than that in the same day matched unexposed nursing period. The mother’s voice stimulation is conducive to maintaining the stability of the heart rate of premature infants. Doheny et al^[44]^ conducted a continuous postnatal monitoring and self-control study on 14 premature infants aged 26 to 32 weeks, showing that maternal voice intervention can reduce the frequency of apnea, and this effect is more significant in infants aged ≥ 33 weeks, which may be related to the integrity of infants’ auditory development. Some studies^[45,46]^ have shown that the mother’s voice, as a preferred auditory stimulus for premature infants, can increase the sense of security of premature infants.

The following limitations of this meta-analysis are worth considering. Firstly, the quality of included RCTs is average. Although the eight studies are all RCT design, there are different degrees of insufficiency in the random methods, allocation hiding, and blind methods, which may lead to selection, implementation and measurement bias. Secondly, it is noted that the included study involved crying of newborn, which should also be an important indicator to evaluate whether the infant’s pain is relieved. We have thought to include the crying of newborn as an important indicator, yet few studies have reported this outcome index, and the relevant data are not enough for the meta-analysis. Future studies should include the crying of newborn for consideration and report this outcome. Thirdly, the start time of each intervention included in RCTs is not completely consistent with the time point of the measurement results, and the data has some heterogeneity. Finally, the literature included in this meta-analysis only includes Chinese and English, which some RCTs reported in other language may be missed for analysis.

## 5. Conclusion

In conclusion, maternal voice stimulation, as a relatively simple and convenient non-drug intervention method, is conducive to maintaining the stability of neonatal process heart rate and blood oxygen saturation, reducing the degree of procedural pain and improving the comfort of neonates. Still, in terms of playing volume, distance between sound source and newborn, starting and ending time of playing, playing content and comparison with other sound intervention means, further studies are needed in the future.

## Author contributions

**Investigation:** Lingwen Jin, Jing Zhang, Xin Yang, Hui Rong.

**Methodology:** Lingwen Jin, Jing Zhang.
